# The cannabinoid CB_1_ receptor and mTORC1 signalling pathways interact to modulate glucose homeostasis in mice

**DOI:** 10.1242/dmm.020750

**Published:** 2016-01-01

**Authors:** Francisco J. Bermudez-Silva, Silvana Y. Romero-Zerbo, Magalie Haissaguerre, Inmaculada Ruz-Maldonado, Said Lhamyani, Rajaa El Bekay, Antoine Tabarin, Giovanni Marsicano, Daniela Cota

**Affiliations:** 1 Unidad de Gestion Clínica Intercentros de Endocrinología y Nutrición, Instituto de Investigación Biomédica de Málaga (IBIMA), Hospital Regional Universitario de Málaga/Universidad de Málaga, Málaga 29009, Spain; 2 Centro de Investigación Biomédica en Red de Diabetes y Enfermedades Metabólicas Asociadas (CIBERDEM), Málaga 29009, Spain; 3 INSERM, Neurocentre Magendie, Physiopathologie de la Plasticité Neuronale, U862, Bordeaux F-33000, France; 4 Université de Bordeaux, Neurocentre Magendie, Physiopathologie de la Plasticité Neuronale, U862, Bordeaux F-33000, France; 5 Service d'endocrinologie, diabétologie, maladies métaboliques et nutrition, Hôpital Haut-Lévêque, Pessac F-33604, France

**Keywords:** Cannabinoids, Insulin secretion, Rapamycin, Rimonabant, Islets, CB_1_, S6K1

## Abstract

The endocannabinoid system (ECS) is an intercellular signalling mechanism that is present in the islets of Langerhans and plays a role in the modulation of insulin secretion and expansion of the β-cell mass. The downstream signalling pathways mediating these effects are poorly understood. Mammalian target of rapamycin complex 1 (mTORC1) signalling is a key intracellular pathway involved in energy homeostasis and is known to importantly affect the physiology of pancreatic islets. We investigated the possible relationship between cannabinoid type 1 (CB_1_) receptor signalling and the mTORC1 pathway in the endocrine pancreas of mice by using pharmacological analysis as well as mice genetically lacking the CB_1_ receptor or the downstream target of mTORC1, the kinase p70S6K1. *In vitro* static secretion experiments on islets, western blotting, and *in vivo* glucose and insulin tolerance tests were performed. The CB_1_ receptor antagonist rimonabant decreased glucose-stimulated insulin secretion (GSIS) at 0.1 µM while increasing phosphorylation of p70S6K1 and ribosomal protein S6 (rpS6) within the islets. Specific pharmacological blockade of mTORC1 by 3 nM rapamycin, as well as genetic deletion of p70S6K1, impaired the CB_1_-antagonist-mediated decrease in GSIS. *In vivo* experiments showed that 3 mg/kg body weight rimonabant decreased insulin levels and induced glucose intolerance in lean mice without altering peripheral insulin sensitivity; this effect was prevented by peripheral administration of low doses of rapamycin (0.1 mg/kg body weight), which increased insulin sensitivity. These findings suggest a functional interaction between the ECS and the mTORC1 pathway within the endocrine pancreas and at the whole-organism level, which could have implications for the development of new therapeutic approaches for pancreatic β-cell diseases.

## Abstract (precis)

**Summary:** Evidence supporting a functional interaction between the endocannabinoid system and the mTORC1 pathway within the endocrine pancreas, which could have implications for the development of new therapeutic approaches for diabetes.

## INTRODUCTION

Endocannabinoids and cannabinoid type 1 (CB_1_) receptors are important players in the regulation of energy homeostasis, having the ability to fine-tune the activity of metabolically relevant tissues, including the hypothalamus, the adipose tissue and the liver (Bensaid et al., 2003; Bermudez-Silva et al., 2012; Bermúdez-Silva et al., 2008; Cota et al., 2003; Lipina et al., 2010; Osei-Hyiaman et al., 2005). Rimonabant, the first-in-class CB_1_ antagonist/inverse agonist was marketed to treat complicated obesity, although central side effects led to its withdrawal later on (reviewed in Bermudez-Silva et al., 2010; Jones, 2008). Rimonabant counteracted the positive energy balance characterizing obese patients by decreasing food intake while inducing energy expenditure and lipolysis, and modulated other peripheral metabolic processes (reviewed in Bermudez-Silva et al., 2012). Among the key metabolic processes modulated by rimonabant, glucose homeostasis is an important one (reviewed in Nogueiras et al., 2008), with two outstanding physiological mechanisms underlying it: insulin sensitivity and insulin release. The latter is controlled by the endocrine pancreas. Nearly all the endocannabinoid system (ECS) components are expressed in the endocrine islets. In particular, CB_1_ receptors are expressed in both alpha (α) and beta (β) cells and their activation is coupled to insulin release, whereas their blockade seems to decrease insulin secretion (reviewed in Li et al., 2011; Doyle, 2011). Interestingly, and underlining the physiological relevance of pancreatic ECS, intra-islet endocannabinoid levels are known to increase after a glucose challenge (Bermúdez-Silva et al., 2008; Kim et al., 2011). Moreover, rimonabant decreases insulin hypersecretion in isolated islets from diabetic rats (Getty-Kaushik et al., 2009), and its chronic administration improves pancreatic function and islet morphology in diabetic rats (Duvivier et al., 2009). Finally, recent evidence points to an important role of CB_1_ receptors in the regulation of expansion of the β-cell mass (Kim et al., 2011, 2012).

The mammalian or mechanistic target of rapamycin complex 1 (mTORC1) is a rapamycin-sensitive multiprotein complex comprising the serine-threonine kinase mTOR and the proteins raptor, PRAS40 and mLST8 (Laplante and Sabatini, 2012). It functions as a nutrient sensor able to integrate signals from growth factors and hormones through the phosphoinositide-3-kinase–protein kinase B/Akt (PI3K-PKB/Akt) pathway. mTORC1 regulates protein synthesis and different aspects of cell growth and metabolism mainly via p70S6 kinase 1 (p70S6K1) and the eukaryotic translation initiation factor 4E-binding protein 1 (4E-BP1) (Laplante and Sabatini, 2012).

Like the ECS, the mTORC1 pathway has also been involved in β-cell physiology (reviewed in Leibowitz et al., 2008). Transient mTORC1 activation leads to increased β-cell/islet size, β-cell mass expansion and insulin production (Blandino-Rosano et al., 2012; Mori and Guan, 2012; Van de Velde et al., 2011; Velazquez-Garcia et al., 2011), whereas chronic mTORC1 activation has rendered conflicting results depending on the nature of the specific activation and/or the experimental model used. mTORC1 overactivation induced by deletion of tuberous sclerosis complex 1 or 2 (TSC1/2) favours β-cell mass expansion and glucose tolerance (Blandino-Rosano et al., 2012; Rachdi et al., 2008), in sharp contrast with other studies in which stimulation of mTORC1 in β cells induced by glucose or IGF-1 led to inhibition of the IRS2/Akt pathway and β-cell apoptosis (Briaud et al., 2005). Conversely, mice deficient for p70S6K1 (*S6K1*^−/−^) are characterized by the lack of the p70S6K1-dependent negative feedback on IRS1/2, leading to enhanced insulin sensitivity and normal glucose levels in diet-induced obesity (Um et al., 2004). Strikingly, mTORC1 inhibition by rapamycin worsens hyperglycaemia and the metabolic state in type 2 diabetes (Fraenkel et al., 2008; Pereira et al., 2012) while reducing islet engraftment and impairing β-cell function (Bell et al., 2003; Bussiere et al., 2006; Zhang et al., 2006). This apparent paradox seems to have been resolved very recently by the demonstration that the deleterious effects of rapamycin might be mediated through mTOR complex 2 (mTORC2) (Barlow et al., 2012; Lamming et al., 2012).

Thus, based on (1) the important role of both the ECS and the mTORC1 pathway in whole-body energy homeostasis, (2) their localization and involvement in key physiological processes within the islets of Langerhans, including insulin secretion and β-cell mass expansion, and (3) previous findings linking both signalling systems in other tissues (Puighermanal et al., 2009; Senin et al., 2013), we hypothesized the existence of a functional link between the ECS and the mTORC1 pathway in the regulation of glucose-stimulated insulin secretion (GSIS) in the islets of Langerhans. Here, we demonstrate that CB_1_ receptor antagonism decreased GSIS in isolated islets of Langerhans from chow-fed lean mice, with this effect requiring a functional mTORC1 pathway. Furthermore, CB_1_ receptor antagonism promoted glucose intolerance *in vivo*, an effect that was counteracted by acute pharmacological blockade of the mTORC1 pathway.

## RESULTS

### Rimonabant decreases glucose-stimulated insulin secretion through CB_1_-dependent and -independent mechanisms

**Fig. 1.**
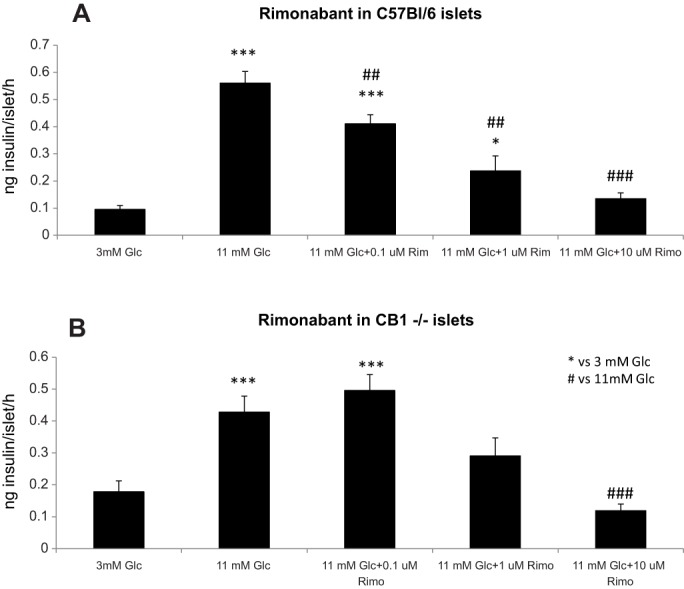
**Rimonabant** **dose-dependently decreases GSIS in isolated islets of Langerhans from C57BL/6 and *CB_1_*^−/−^ mice.** (A) 0.1, 1 and 10 µM rimonabant decreased GSIS in islets from C57BL/6 mice, when compared to vehicle-treated, glucose-stimulated wells (11 mM Glc), in a dose-dependent manner. (B) By contrast, only 10 µM rimonabant statistically decreased GSIS in islets from *CB_1_*^−/−^ mice. Data from three independent experiments in C57BL/6 mice and in *CB_1_*^−/−^ mice, islets from 2-3 animals each experiment. *n*=5-6 wells each experimental condition. ***P<0.001 and *P<0.05, vs 3 mM Glc; ^###^*P*<0.001 and ^##^*P*<0.01, vs 11 mM Glc; one-way ANOVA.

We performed *in vitro* dose-response static secretion experiments in isolated islets from adult male C57BL/6, *CB_1_*^−/−^ mice and their wild-type littermates (*CB_1_*^+/+^), and *S6K1^−/−^* mice and their wild-type littermates (*S6K1^+/+^*). No changes in insulin secretory capacity were detected between genotypes in neither CB_1_ nor S6K1 mice (data not shown). Dose-response experiments with rimonabant were performed in C57BL/6, *CB_1_*^+/+^ and *CB_1_*^−/−^ mice. Rimonabant decreased GSIS in a dose-dependent manner in C57BL/6 mice (Fig. 1A). As compared to 11 mM, vehicle-treated wells, 0.1 µM, 1 µM and 10 µM rimonabant significantly decreased GSIS in a dose-dependent manner. Similar results were obtained in *CB_1_*^+/+^ mice (Fig. S1A). Conversely, only the highest tested dose of rimonabant (10 µM) was able to statistically decrease GSIS in islets from *CB_1_*^−/−^ littermates, whereas 1 µM showed a tendency to decrease insulin secretion (Fig. 1B), implying that a 0.1 µM dose of rimonabant requires CB_1_ to inhibit insulin secretion and higher doses act on the pancreatic islets in a CB_1_-independent manner.

### Pharmacological blockade of CB_1_ receptors requires functional p70S6K1 to decrease GSIS

**Fig. 2.**
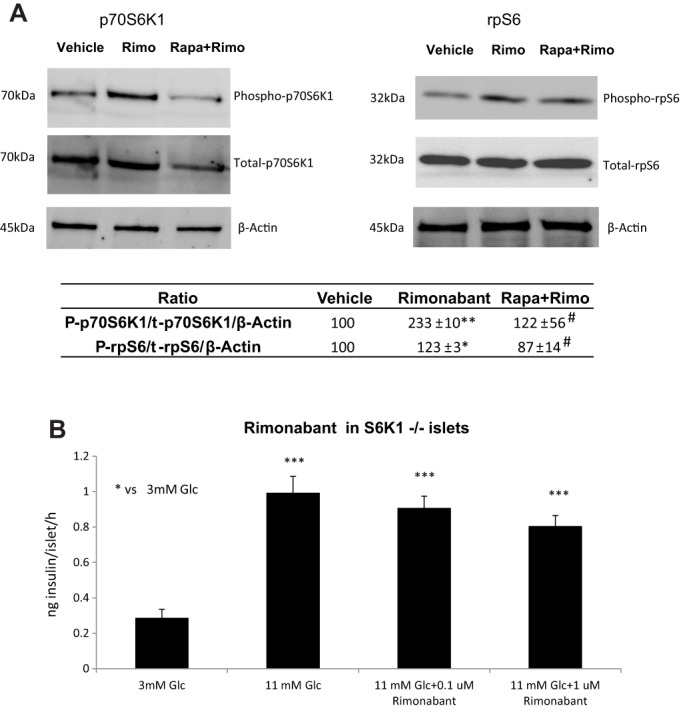
**Rimonabant action on GSIS is dependent on the mTORC1 pathway and it phosphorylates p70S6K1 and rpS6 in a rapamycin-sensitive way.** (A) CB_1_ antagonism by rimonabant increases *in vitro* phosphorylation of p70S6K1 and rpS6 in isolated islets of Langerhans, and this effect is prevented by rapamycin pre-incubation. Total-p70S6K1, total-rpS6 and β-actin were used as reference proteins. The image is representative of three independent experiments per protein. Table: immunoreactivity was measured by using ImageJ software and values are expressed as percentage of signal intensity in vehicle condition. *P<0.05, **P<0.01 vs vehicle; ^#^*P*<0.05 vs rimonabant; Student's *t*-test. (B) Antagonism of CB_1_ with rimonabant had no effect on GSIS in isolated islets from *S6K1*^−/−^ mice. Data from three independent experiments. Islets from 2-3 animals each experiment. *n*=5-6 well each experimental condition. ***P<0.001 vs 3 mM Glc; one-way ANOVA.

In a first attempt to test the functional interaction between CB_1_ receptor signalling and the mTORC1 pathway in the islets, we verified whether blockade of CB_1_ receptor affected phosphorylation levels of p70S6K1 and of ribosomal protein S6 (rpS6), two major downstream targets of mTORC1 classically used as a readout of mTORC1 activity (Laplante and Sabatini, 2012). Incubation of C57BL/6 islets with 0.1 µM rimonabant for 15 min in 11 mM glucose led to an increase in the phosphorylation of p70S6K1 and rpS6 when compared to both total protein and β-actin levels (Fig. 2A). Moreover, pre-incubation of islets with 3 nM rapamycin prevented the rimonabant-induced increase of p70S6K1 and rpS6 phosphorylation. These data therefore suggest that rimonabant, used at the dose shown to affect insulin secretion in a CB_1_-dependent manner, activated the mTORC1 pathway in pancreatic islets.

To further determine whether phosphorylation of p70S6K1 represented a crucial step in determining the action of rimonabant on GSIS, we tested the ability of the CB_1_ receptor antagonist to modulate GSIS in islets of male *S6K1*^−/−^ mice. *In vitro* static secretion experiments were carried out on *S6K1*^−/−^ islets by treating them with 0.1 and 1 µM rimonabant. However, neither dose affected GSIS in isolated *S6K1*^−/−^ islets, whereas 1 µM rimonabant decreased GSIS in *S6K1*^+/+^ islets (Fig. 2B and Fig. S1B).

### Pharmacological blockade of mTORC1 impairs the rimonabant-induced decrease in GSIS

**Fig. 3.**
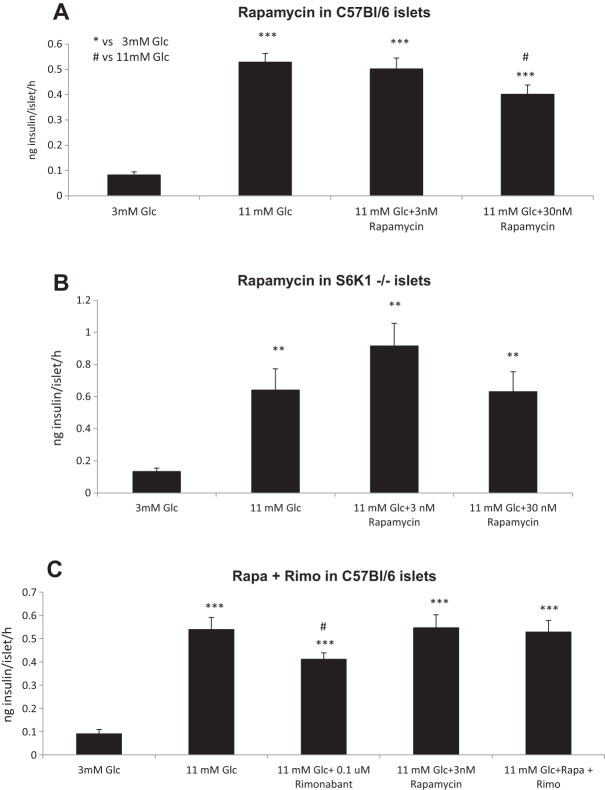
**Rapamycin decreases GSIS in isolated islets of Langerhans through mTORC1 and prevents the rimonabant-induced decrease in GSIS.** (A) 30 nM rapamycin decreased GSIS in isolated islets from C57BL/6 mice when compared to vehicle-treated, glucose-stimulated wells (11 mM Glc). (B) Rapamycin had no effect at these doses in isolated islets from *S6K1*^−/−^ mice. Data from four independent experiments, two of each genotype, islets from 2-3 animals each experiment. *n*=5-6 well each experimental condition. **P<0.01 and ***P<0.001 vs 3 mM Glc, respectively; ^#^*P*<0.05 vs 11 mM Glc; one-way ANOVA. (C) 0.1 µM rimonabant decreased GSIS in C57BL/6 isolated islets, whereas 3 nM rapamycin per se had no effect. However, 3 nM rapamycin pre-treatment prevented the rimonabant-induced decrease in GSIS. Data from two independent experiments, islets from 2-3 animals each experiment. *n*=5-6 well each experimental condition. ***P<0.001 vs 3 mM Glc; ^#^*P*<0.05 vs 11 mM Glc; one-way ANOVA.

In order to explore the effect of the mTORC1 inhibitor rapamycin on GSIS, and the specificity of the doses here assayed over mTORC1, we performed *in vitro* static secretion experiments on islets from male C57BL/6 and *S6K1*^+/+^ mice. Treatment of islets with rapamycin significantly decreased GSIS from the 11 mM glucose condition (Fig. 3A and Fig. S1C), whereas it did not have any effect in islets from *S6K1*^−/−^ mice (Fig. 3B), suggesting that a functional mTORC1 pathway and p70S6K1 in particular are required for the action of rapamycin on GSIS. We therefore assessed the possible interaction between specific doses of rapamycin and rimonabant on GSIS using islets from male C57BL/6 mice. Islets were incubated with 3 nM of rapamycin or vehicle and further treated with 0.1 µM rimonabant or vehicle. Fig. 3A and C show that 3 nM rapamycin had no effect per se on GSIS in islets from C57BL/6 mice, but it prevented the effect of rimonabant on GSIS.

### Rimonabant induces glucose intolerance *in vivo* through CB_1_ receptors

**Fig. 4.**
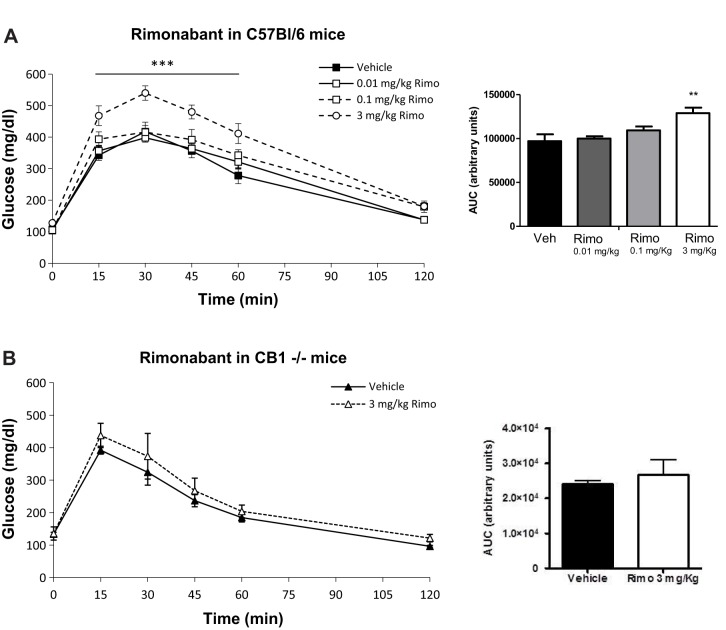
**Rimonabant induces glucose intolerance in mice through CB_1_ receptors.** (A) Glucose tolerance test (GTT) in C57BL/6 mice pre-treated with increasing doses of rimonabant. Rimonabant was injected intraperitoneally (i.p.) 30 min before glucose challenge. ANOVA with repeated measures shows significant differences between the glucose curve for 3 mg/kg rimonabant and vehicle (***P<0.001). Insert: quantification of glucose AUC in vehicle- and rimonabant-treated mice. **P<0.01, 3 mg/kg rimonabant versus vehicle; one-way ANOVA. Two independent experiments, *n*=7-8 mice each experimental condition. (B) 3 mg/kg rimonabant did not affect plasma glucose levels during a GTT in *CB_1_*^−/−^ mice. Insert: quantification of glucose AUC. Two independent experiments, *n*=4-6 mice each experimental condition.

To study the effects of the CB_1_-receptor–mTORC1 interaction on glucose homeostasis *in vivo*, we performed a glucose tolerance test (GTT) after administration of rimonabant or rapamycin. First, C57BL/6 mice were treated with different doses of rimonabant 30 min before the GTT: 3 mg/kg (mg/kg body weight) rimonabant increased glucose plasma levels before the glucose load (vehicle: 107±5 mg/dl; 0.01 mg/kg rimonabant: 104±6 mg/dl; 0.1 mg/kg rimonabant: 105±8 mg/dl; and 3 mg/kg rimonabant: 128±6 mg/dl, *P*<0.05). After the administration of glucose, mice treated with the highest dose of rimonabant (3 mg/kg) showed significantly higher glucose plasma levels as compared to vehicle-treated mice as well as to the 0.1 mg/kg and 0.01 mg/kg rimonabant-treated groups (Fig. 4A). Accordingly, glucose area under the curve (AUC) quantification was higher in 3 mg/kg rimonabant pre-treated mice than in all the other groups (Fig. 4A, insert). Importantly, plasma insulin levels in 3 mg/kg rimonabant-treated mice were lower than in vehicle-treated controls 15 min after glucose overload (vehicle: 0.794±0.109 ng/ml; 3 mg/kg rimonabant: 0.468±0.067 ng/ml, *P*<0.05). We then assessed the CB_1_ receptor involvement in rimonabant-induced glucose intolerance by performing similar experiments on *CB_1_*^+/+^ and *CB_1_*^−/−^ littermates. The dose of 3 mg/kg rimonabant was effective in increasing plasma glucose levels in *CB_1_*^+/+^ mice after a glucose load (data not shown), whereas this effect was absent in *CB_1_*^−/−^ littermates (Fig. 4B). The insert in Fig. 4B shows no changes in AUC quantification between vehicle- and 3 mg/kg rimonabant-treated mice.

### *In vivo* pharmacological blockade of the mTORC1 pathway counteracts rimonabant-induced glucose intolerance

**Fig. 5.**
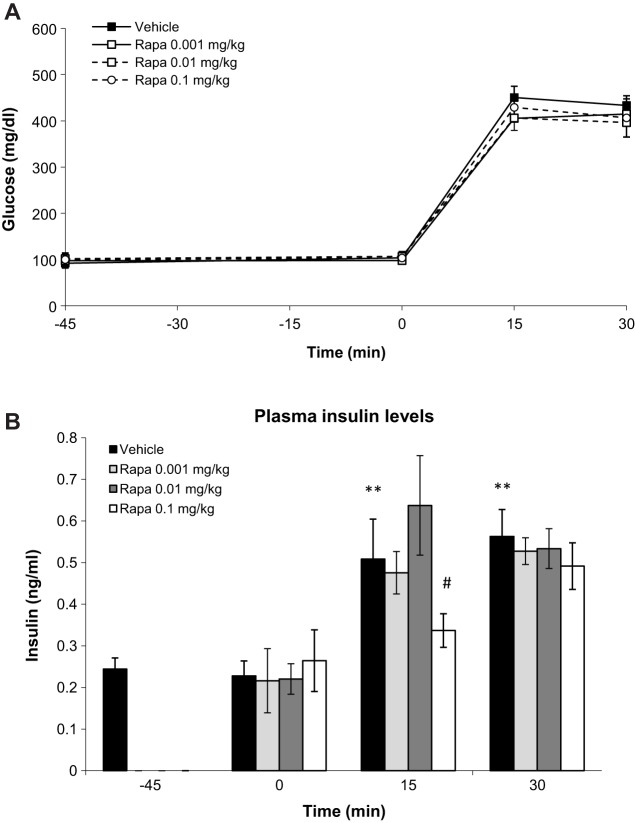
**Rapamycin does not affect glucose tolerance *in vivo* in the range 0.001-0.1 mg/kg but decreases plasma insulin levels at 0.1 mg/kg.** (A) Glucose levels were monitored at time points −45 (basal), 0, 15 and 30 min after glucose load in C57BL/6 mice receiving rapamycin at 0.001, 0.01 or 0.1 mg/kg. (B) Effect of different doses of rapamycin on plasma insulin before (−45 min.) and 0, 15 and 30 min after glucose challenge in C57BL/6 mice. **P<0.01 versus vehicle at time point 0 min; ^#^*P*<0.05 versus vehicle at time point 15 min; one-way ANOVA. Two and three independent experiments panel A and B, respectively, *n*=5-8 mice each experimental condition.

**Fig. 6.**
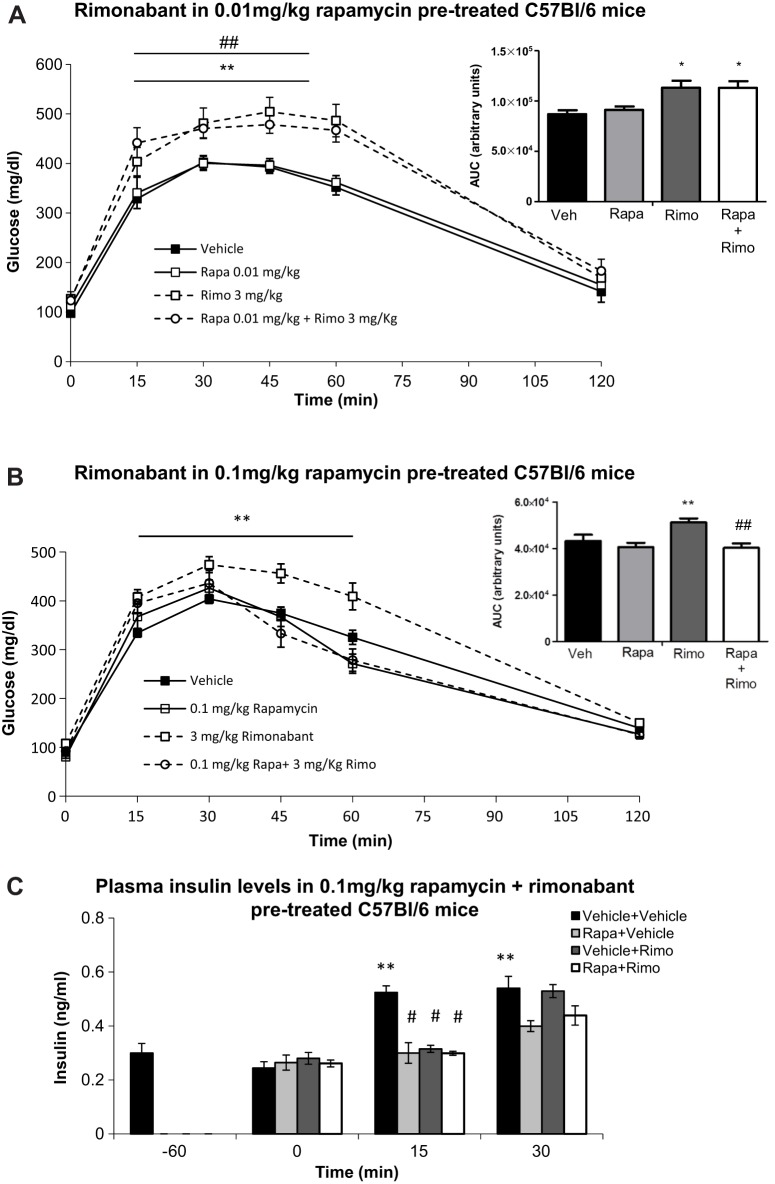
**0.01 mg/kg rapamycin does not prevent the rimonabant-induced decrease in glucose tolerance *in vivo*, whereas 0.1 mg/kg does.** (A) GTT in C57BL/6 mice treated with vehicle, rapamycin at 0.01 mg/kg, rimonabant at 3 mg/kg and rapamycin plus rimonabant (rapa+rimo). ANOVA with repeated measures shows significant differences between the glucose curve for 3 mg/kg rimonabant and vehicle (**P<0.01) and between the glucose curve for rapa+rimo and vehicle (^##^*P*<0.01). Insert: quantification of glucose AUC. *P<0.05 versus vehicle; one-way ANOVA. Two independent experiments, *n*=7-8 mice each experimental condition. (B) GTT in C57BL/6 mice treated with vehicle, rapamycin at 0.1 mg/kg, rimonabant at 3 mg/kg and rapa+rimo. ANOVA with repeated measures shows significant differences between the glucose curve for 3 mg/kg rimonabant and vehicle (**P<0.01). Insert: quantification of glucose AUC. **P<0.01, rimonabant versus vehicle, ^##^*P*<0.01 rapa+rimo versus rimonabant; one-way ANOVA. Two independent experiments, *n*=7-10 mice each experimental condition. (C) Plasma insulin levels in C57BL/6 mice before treatments (−60 min) and 0, 15 and 30 min after vehicle, rapamycin at 0.1 mg/kg, rimonabant at 3 mg/kg or rapa+rimo. **P<0.05 versus vehicle at time 0; ^#^*P*<0.05 versus vehicle at time 15; one-way ANOVA. Three independent experiments, *n*=7-8 mice each experimental condition.

Having found that rimonabant caused glucose intolerance *in vivo* in lean animals, we went on to assess whether this action could be counteracted by rapamycin, as already demonstrated *in vitro* for the regulation of GSIS. First, we performed a GTT, collecting samples for plasma insulin measurements at −45 (basal), 0 (just before glucose overload), 15 and 30 min after the administration of glucose, and assessed the effect of low doses of rapamycin on glucose levels at these time points (Fig. 5A). At the doses tested, rapamycin did not alter glucose tolerance (Fig. 5A). However, 0.1 mg/kg rapamycin decreased insulin plasma levels at 15 min after the glucose load (Fig. 5B). Based on these results, the highest non-effective dose of rapamycin (0.01 mg/kg) and the insulin-acting dose of rapamycin (0.1 mg/kg) were subsequently combined with rimonabant administration. As expected, CB_1_ receptor antagonism alone increased plasma glucose levels after the glucose load (Fig. 6A and insert), whereas rapamycin alone at the dose of 0.01 mg/kg had no effect. Similarly, when combined to rimonabant, this dose of rapamycin was unable to counteract rimonabant action (Fig. 6A and insert). Conversely, pre-treatment with 0.1 mg/kg rapamycin prevented the rimonabant-induced increase in glucose levels (Fig. 6B and insert). Insulin plasma levels were measured before injections (−60), and 0, 15 and 30 min after the glucose load. Fig. 6C shows that all treatments significantly decreased plasma insulin levels at 15 min, suggesting that rapamycin exerts extra-pancreatic effects that are in turn involved in counteracting rimonabant-induced glucose intolerance *in vivo*.

### Rapamycin counteracts rimonabant actions *in vivo* by increasing peripheral insulin sensitivity

**Fig. 7.**
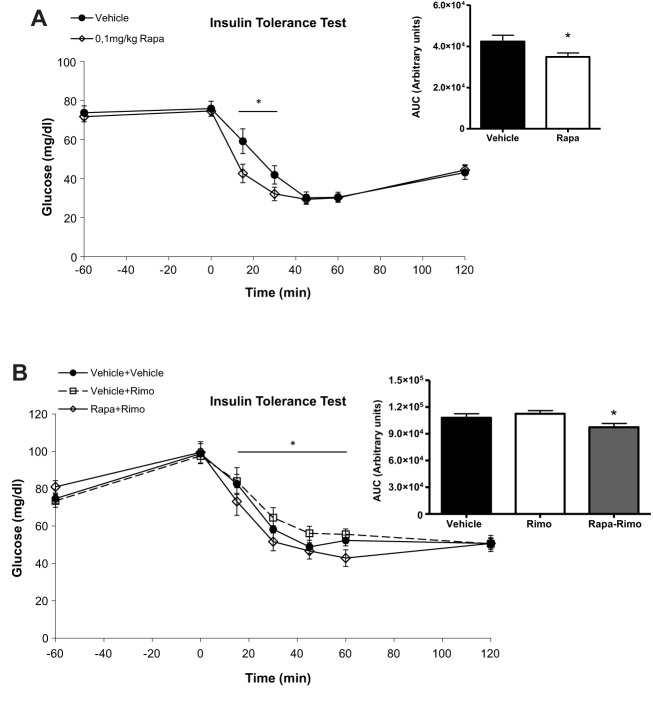
**0.1 mg/kg rapamycin increases insulin sensitivity alone and in combination with rimonabant**. (A) ITT in C57BL/6 mice treated with vehicle and 0.1 mg/kg rapamycin. ANOVA with repeated measures shows significant differences between the glucose curve for rapamycin and vehicle (*P<0.05). Insert: quantification of glucose AUC in vehicle and rapamycin-treated mice. *P<0.05; one-way ANOVA. Two independent experiments, *n*=7-8 mice each experimental condition. (B) ITT in C57BL/6 mice treated with vehicle, rimonabant at 3 mg/kg and rapamycin at 0.1 mg/kg+rimonabant at 3 mg/kg. ANOVA with repeated measures shows significant differences between the glucose curve for rimonabant and rapa+rimo (*P<0.05). Insert: quantification of glucose AUC in vehicle, rimonabant- and rapa+rimo-treated mice. *P<0.05 versus rimonabant; one-way ANOVA. Two independent experiments, *n*=7-8 mice each experimental condition.

We wanted to assess whether changes in insulin sensitivity were underlying the rapamycin-reverting effects of rimonabant actions on glucose homeostasis. For this purpose, an insulin tolerance test (ITT) was performed in fasted mice pre-treated with 0.1 mg/kg rapamycin. Fig. 7A shows that this dose of rapamycin decreased glucose levels at time points 15 and 30 min after insulin injection, also decreasing the AUC (Fig. 7A insert). An ITT was also performed on fasted mice pre-treated with rimonabant alone (3 mg/kg) or receiving both rapamycin (0.1 mg/kg) and rimonabant (3 mg/kg). Fig. 7B shows that rimonabant alone did not induce changes in insulin sensitivity, whereas pre-treatment with 0.1 mg/kg rapamycin increased insulin sensitivity (Fig. 7B), with the AUC being significantly decreased in rapamycin+rimonabant-injected mice versus both vehicle- and rimonabant-injected mice (Fig. 7B insert).

## DISCUSSION

In this manuscript, we present evidence that cannabinoid CB_1_ receptors and the mTORC1 pathway interact in the islets of Langerhans to modulate GSIS. Furthermore, systemic pharmacological blockade of these two metabolic signalling systems involved in energy homeostasis and nutrient sensing reveals a functional interaction in the regulation of glucose homeostasis.

Pharmacological modulation of CB_1_ receptors in the islets modulates GSIS (for recent reviews see Li et al., 2011; Doyle, 2011) and β-cell mass (Duvivier et al., 2009; Getty-Kaushik et al., 2009; Kim et al., 2011, 2012). Thus, it looks likely that both GSIS and β-cell mass expansion are cellular processes intrinsically modulated by endocannabinoids. However, there are conflicting data in the literature regarding which cell types express (or not) CB_1_ within the endocrine pancreas and in which way CB_1_ activation alters GSIS. Adding more controversy to this issue, rimonabant has been shown to act not only through CB_1_ receptors but also through GPR55 (Kapur et al., 2009; Lauckner et al., 2008; Yin et al., 2009), and GPR55 is expressed in the endocrine pancreas, with its activation increasing GSIS (Romero-Zerbo et al., 2011; McKillop et al., 2013). Our results on C57BL/6, *CB_1_*^+/+^ and *CB_1_*^−/−^ islets show that rimonabant promotes both CB_1_-dependent and CB_1_-independent decreases of GSIS, with doses ranging from 0.1 to 1 µM requiring action on CB_1_ receptors. Indeed, these doses decreased plasma insulin levels and induced glucose intolerance in C57BL/6 and *CB_1_*^+/+^, but not in *CB_1_*^−/−^ littermates.

In previous studies, we have investigated glucose tolerance in chow-fed rats using another CB_1_ antagonist, AM251, and this drug was found to increase glucose tolerance at low doses (0.01-0.2 mg/kg), whereas it had no effect at higher doses (1 mg/kg) (Bermudez-Silva et al., 2007; Bermúdez-Siva et al., 2006). A couple of reasons could account for the different effects observed with AM251. First, several investigations have shown that AM251 is a potent agonist of GPR55 (Kapur et al., 2009), and GPR55 is expressed in rat islets, as previously mentioned (Romero-Zerbo et al., 2011), which suggests that some of the previously published findings using AM251 could be partly due to GPR55 activation. Second, it has been reported that there is some degree of species-specific expression for CB_1_, CB_2_ and GPR55 (Li et al., 2011; Romero-Zerbo et al., 2011), with CB_1_ and GPR55 being more intensely expressed in rat than in mouse β cells. Of note, in the present study, we validated our findings in *CB_1_*^−/−^ mice, showing that rimonabant-induced glucose intolerance is mediated through CB_1_ receptors. Indeed, lower plasma insulin levels were detected in mice injected with 3 mg/kg rimonabant when compared to vehicle-injected mice, suggesting that, at least in part, glucose intolerance was due to decreased insulin secretion, probably as a consequence of the inhibitory effects of rimonabant on islet function. Taken together, these results indicate that CB_1_ receptors in the islets of chow-fed lean mice are important in the modulation of whole-body glucose homeostasis.

With the present results we expand the knowledge about the downstream mechanisms being recruited by CB_1_ receptors in the islets to modulate GSIS. In fact, both genetic and pharmacological blockade of mTORC1 abolish CB_1_-mediated rimonabant action on GSIS. Interestingly, a dose of rapamycin lacking effect on GSIS is sufficient to block rimonabant action, whereas a higher dose decreases GSIS. This evidence suggests that rapamycin acts simultaneously on different targets to modulate GSIS. Whereas a low dose inhibits the rimonabant-induced effect on GSIS, thus unmasking the dependence of rimonabant action on an intact mTORC1 pathway, higher doses of rapamycin decrease GSIS. Rapamycin has been found to inhibit mTORC2 (Barlow et al., 2012; Lamming et al., 2012) and to cause insulin resistance (Lamming et al., 2012). However, doses reported to induce insulin resistance were higher than the ones used in our study and they were chronically administered. By contrast, we demonstrated that rapamycin administered acutely at 0.1 mg/kg, although decreasing insulin levels, increased insulin sensitivity. Although we have not measured rapamycin plasma levels in our animals, according to previous findings (Guba et al., 2005), 0.1 mg/kg rapamycin should be leading to around 20-30 nM of the compound in the plasma, a dose that we have found to decrease GSIS through mTORC1. Thus, the effect of rapamycin on GSIS is dependent on mTORC1, although further studies are needed in order to unequivocally establish that action on mTORC2 or other molecular pathways is not required.

A CB_1_-acting dose of rimonabant increased phosphorylation of both p70S6K1 and rpS6, downstream targets of the mTORC1 pathway, and decreased GSIS. However, the mechanisms linking S6K1/rpS6 phosphorylation and GSIS are unclear at present. mTORC1 is an important player in islet physiology, especially in the control of β-cell mass (Pende et al., 2000), which has also been shown to be involved in the pathophysiology of type 2 diabetes (Leibowitz et al., 2008). Although β-cell mass investigation was beyond the scope of our work, it is noteworthy that Kim and colleagues have shown that CB_1_ blockade in islets is coupled to increased insulin receptor activation through impaired Gαi/o-coupled inhibition, Akt phosphorylation and increased β-cell mass (Kim et al., 2011, 2012). Given that Akt is a well-known activator of mTORC1, the rimonabant-induced increase in mTORC1 activation might be mediated by the Akt pathway. Likewise, given the central role of mTORC1 in modulating β-cell mass (Rachdi et al., 2008; Leibowitz et al., 2008), our results suggest that the previously reported CB_1_-dependent β-cell mass expansion (Kim et al., 2011) could involve mTORC1. Rimonabant has been described to decrease plasma insulin levels and insulin hyper-secretion from isolated islets of diabetic Zucker rats (Duvivier et al., 2009; Getty-Kaushik et al., 2009). Furthermore, histo-morphological analysis of pancreas from rimonabant-treated Zucker rats has suggested a protective role of CB_1_ blockade on islet integrity (Duvivier et al., 2009). Consequently, beyond the important role of islet CB_1_ receptors in physiology, rimonabant treatment in diabetes improves islet function by mechanisms that are still not well understood. Our findings suggest that mTORC1 activation could be one of these mechanisms, although additional experiments on diabetic animals need to be carried out in the future.

**Fig. 8.**
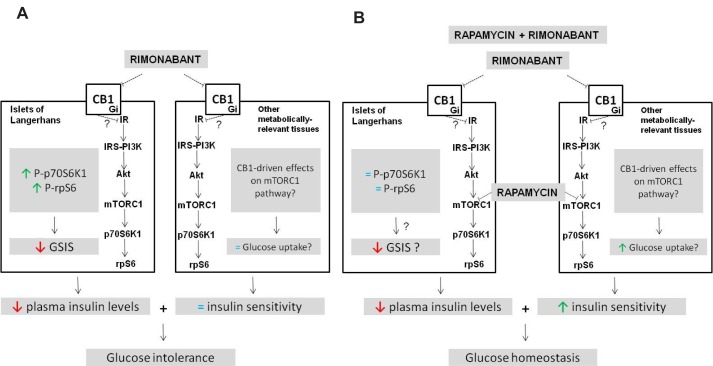
**Schematic diagram illustrating the proposed mechanism of action for CB_1_-mTORC1 interaction in islets and its physiological relevance *in vivo*.** (A) Rimonabant antagonizes CB_1_ receptors in islets and, probably by uncoupling the Gi subunit at the insulin-response element (IRS) of the insulin receptor or another related growth-factor receptor, activates the Akt-mTORC1 pathway, increasing phosphorylation of p70S6K1 and of rpS6. This activation decreases GSIS and consequently plasma insulin levels, leading to glucose intolerance. No changes in peripheral insulin sensitivity were observed with rimonabant (= insulin sensitivity). (B) Rapamycin prevents rimonabant-mediated activation of the Akt-mTORC1 pathway, thus preventing rimonabant-dependent decrease in GSIS; however, insulin plasma levels remain decreased as a consequence of rimonabant-unrelated rapamycin actions, probably also mediated through decreased GSIS. In parallel, rapamycin increases insulin sensitivity by acting at other peripheral tissues, likely by increasing glucose uptake, with the net effect being an appropriate glucose homeostasis. Gi, inhibitory G protein; IR, insulin receptor; IRS, insulin receptor substrate; PI3K, phosphoinositide 3-kinase.

To study the *in vivo* relevance of the interaction between the ECS and mTORC1 signalling on glucose homeostasis, we first analyzed glucose tolerance and insulin plasma levels in rapamycin-injected mice. We found no effect on glucose tolerance when rapamycin was used in the range of 0.001-0.1 mg/kg, but the highest dose decreased plasma insulin levels. Then, to study the putative interaction with CB_1_-dependent signalling, we combined the intraperitoneal administration of rapamycin with rimonabant. Rapamycin at 0.1 mg/kg counteracted rimonabant action on glucose responses during a GTT. However, both rimonabant and 0.1 mg/kg rapamycin decreased plasma insulin levels, with their combination not altering plasma insulin levels further. This latter result implies that rapamycin prevents rimonabant-induced glucose intolerance by increasing insulin sensitivity, as was shown by an ITT. Thus, these pharmacological *in vivo* experiments suggest that the pancreatic interaction between CB_1_ signalling and the mTORC1 pathway has a whole-body impact on glucose homeostasis (see Fig. 8). However, additional extra-pancreatic effects of rimonabant on glucose homeostasis cannot be ruled out and those of rapamycin are evident. In fact, rapamycin at the dose of 0.1 mg/kg has insulin-sensitising properties in mice, an effect that could be related to increased glucose uptake in insulin target tissues, such as the muscle or the adipose tissue. Administration of rapamycin *in vivo* has generally led to insulin resistance and glucose intolerance (Fraenkel et al., 2008; Rachdi et al., 2008). In the muscle, rapamycin did not improve either insulin sensitivity nor glucose tolerance (Miller et al., 2008), although higher doses (5 mg/kg) that likely targeted mTORC2 were used. In isolated human subcutaneous and omental adipocytes, *ex vivo* incubation with low doses of rapamycin (10 nM) impaired insulin signalling and glucose uptake (Pereira et al., 2012), but the extent to which this effect contributes to peripheral insulin sensitivity in humans is unknown. Our findings instead suggest that low doses of rapamycin, by specifically inhibiting mTORC1, could be beneficial in pathological conditions where an excessive insulin secretion and signalling is present, such as in insulin resistance (Fig. 8).

Thus, we propose a molecular model by which CB_1_ receptor antagonism in islets leads to decreased GSIS through mTORC1 activation. This in turn would decrease plasma insulin levels without altering insulin sensitivity and hence inducing glucose intolerance (Fig. 8A). On the other hand, mTORC1 blockade would prevent the rimonabant-induced decrease in GSIS, although it decreases GSIS per se, while increasing peripheral insulin sensitivity, thus maintaining glucose homeostasis (Fig. 8B). Our experiments do not allow the determination of whether CB_1_ receptors are functionally coupled to mTORC1 in metabolic processes at other peripheral tissues, but this possibility cannot be ruled out and will need to be investigated in future studies. Unravelling the coupling between CB_1_ receptors and the mTORC1 pathway also deserves more experimental work, but, based on the published literature, it is tempting to speculate that rimonabant is blocking the inhibitory action of the CB_1_-Gi protein on phosphorylation of insulin receptors (or other growth-factor receptors) present in islets and in turn phosphorylation of IRS1/2. In fact, the inhibitory actions of endocannabinoids on insulin receptor phosphorylation in islets have been reported previously (Kim et al., 2011).

Overall, our results support the notion that CB_1_ and mTORC1 signalling share common molecular circuits in the endocrine pancreas so as to modulate insulin secretion and that this interaction is relevant at the whole-body level to modulate glucose homeostasis. These observations open new research avenues regarding the potential therapeutic value of strategies exploiting this functional interaction in diabetes. The next steps should include an in-depth exploration of the CB_1_-mTORC1 interaction in the modulation of both glucose-stimulated insulin secretion and β-cell mass expansion both in human islets and in animal models of type 2 diabetes.

## MATERIALS AND METHODS

### Animals and drugs

Male C57BL/6 mice were purchased from Janvier (Janvier SAS, Le Genest-Saint-Isle, France). Male *S6K1*^−/−^ and *S6K1*^+/+^ mice, and male *CB_1_*^−/−^ and *CB_1_*^+/+^ littermates, were also used. The *S6K1*^−/−^ mouse strain, a kind gift of Dr S. Kozma and Dr G. Thomas (University of Cincinnati and IDIBELL Barcelona), was generated and genotyped as previously described (Shima et al., 1998; Um et al., 2004). It was out-crossed at least twice to C57BL/6J mice upon arrival at the Neurocentre Magendie, Bordeaux, France and was maintained on heterozygous breeding. The *CB_1_*^−/−^ strain was generated and genotyped as previously reported (Marsicano et al., 2002). Mice were maintained on a 12-h light-dark cycle (lights off at 13:00 h) with *ad libitum* access to pelleted chow and water, unless otherwise specified. All experiments were performed on male mice aged 11-15 weeks, fed a standard normocaloric chow (SAFE A04C, Augy, France) with the following nutritional composition in %: Glucids 60, Proteins 16, Lipids 3, Moisture 12, Minerals 5, Fibres 4. Experiments were conducted in strict compliance with the European Union recommendations (2010/63/EU). The experimental procedures were approved by the French Ministry of Agriculture and Fisheries (animal experimentation authorization no. 3309004) and by the ethic committee of the University of Malaga (animal experimentation authorization no. 2012-0002A). Animals were sacrificed by cervical dislocation. The number of animals used in each experiment is specified in the figure legends.

Rimonabant was purchased from Cayman Chemicals (Ann Arbor, MI, USA) and diluted in 0.1% DMSO or ethanol in saline. Rapamycin was purchased from Merck (Merck KGaA, Darmstadt, Germany) and diluted in 0.1% ethanol in saline.

### Islet isolation and *in vitro* GSIS experiments

Pancreatic islets were isolated by the collagenase digestion method (Tudurí et al., 2009). Briefly, pancreas was inflated with Hanks solution containing 0.33 mg/ml of collagenase (Sigma-Aldrich, St Louis, MO, USA), 5.6 mM glucose and 1% bovine serum albumin, pH 7.35, removed and kept at 37°C for 6-9 min. After tissue digestion and exocrine removal by three consecutive washes, the islets were manually collected, under a binocular magnifier, as in Tudurí et al., 2009. Islets were left recovering from digestion by culturing for 20-24 h in RPMI-1640 medium containing 11 mM glucose (Invitrogen, CA, USA) and supplemented with 2 mM glutamine, 200 IU/ml penicillin, 200 μg/ml streptomycin and 8% fetal bovine serum stripped with charcoal-dextran (Invitrogen). For static incubation experiments, islets were first incubated for 2 h at 37°C in 3 ml Krebs-bicarbonate buffer solution (in mM): 14 NaCl, 0.45 KCl, 0.25 CaCl_2_, 0.1 MgCl_2_, 2 HEPES and 3 glucose, equilibrated with a mixture of 95% O_2_:5% CO_2_, pH 7.4. Groups of five size-matched islets were transferred to 24-well plate wells with 0.5 ml fresh buffer containing either one of the following solutions: 3 mM glucose, 11 mM glucose plus vehicle (0.4% DMSO), or 11 mM glucose plus the diluted drug to be tested, and further incubated for 1 h. Rimonabant and rapamycin were diluted in DMSO to a final working dilution containing 0.4% DMSO. At the end of the incubation, bovine albumin was added to each well to a final concentration of 1%, and the plate was put at 4°C for 15 min to stop insulin secretion. Next, the media was collected and stored at −20°C for subsequent measurement of insulin content by ELISA (Mercodia, Uppsala, Sweden), according to the manufacturer's instructions. Insulin secretion was expressed as ng insulin per islet and per hour of incubation.

### Western blot

In order to investigate protein expression of phosphorylated p70S6K1, total-p70S6K1 and β-actin in isolated islets after rimonabant or rapamycin plus rimonabant exposure, groups of 150 islets cultured for 20-24 h in RPMI-1640 medium (see above) were transferred to 5-cm Petri dishes and pre-treated for 15 min with 3 nM rapamycin or vehicle and then treated for 15 min with either 0.1 µM rimonabant or vehicle. Islets were then collected, washed twice with cold PBS and transferred to pre-chilled Precellys tubes (Bertin Technologies, Montigny le Bretonneux, France) containing 150 µl ice-cold RIPA lysis buffer plus 1 mmol/l sodium orthovanadate, 50 mmol/l sodium fluoride, 50 µl Complete Protease Inhibitor Cocktail (Roche Diagnostics, Mannheim, Germany) and 50 µl PhosphoStop solution (Roche). Islets were homogenized using the Precellys homogenizer at 2300 ***g***, 2×30 min with a 10 s interval. Next, the tubes were centrifuged at 10,000 ***g*** for 10 min at 4°C, supernatants were removed and centrifuged again before snap freezing the cell lysates. Cell lysates (12 µg each lane) were subjected to SDS-PAGE on 8% polyacrylamide gels and electrotransferred on a PVDF membrane. Membranes were then blocked for 1 h in TBS-Tween (TBST: 50 mmol/l Tris-HCl, pH 7.5, 0.15 mol/l NaCl and 0.1% Tween) containing 5% skimmed milk and probed for 16 h at 4°C in TBST, 5% skimmed milk with 1/1000 dilution of the antibody [anti-phospho-p70S6K1 thr389 (EMD Millipore Corporation, Billerica, MA, USA), anti-phospho-rpS6 Ser240/244 (Cell Signaling, Beverly, MA, USA), anti-total-p70S6K1 (EMD Millipore Corporation), anti-total-rpS6 (Cell Signaling), anti-β-actin (1/10,000; Sigma-Aldrich)]. Detection of proteins was performed using horseradish-peroxidase-conjugated secondary antibodies and an enhanced chemiluminescence reagent (Amersham Biosciences, Little Chalfont, UK). Quantification was carried out using ImageJ (National Institutes of Health, USA).

### Glucose tolerance test (GTT) and insulin tolerance test (ITT)

Glucose tolerance and insulin tolerance was investigated in 11- to 12-week-old, 12- to 14-h-fasted mice, treated before the glucose or insulin injection by intraperitoneal administration of rapamycin and/or rimonabant. Rapamycin or its vehicle was injected 45 min before glucose (30 min before glucose in experiments only administering rapamycin) and rimonabant or its vehicle was injected 30 min before glucose. The mice were moved to the experimental room 30 min before the first injection. The GTT was carried out by injecting an intraperitoneal glucose load of 2 g/kg body weight diluted in saline and the ITT by injecting 0.75 IU/kg body weight prepared in the same diluent. Glucose was determined using a commercial glucometer (Accu-check, Roche Diagnostic, Barcelona, Spain) from tail blood drops before the first injection (−60 min or −45 min), just before glucose (0 min) and 15, 30, 45, 60 and 120 min after glucose administration. To measure plasma insulin, every 2 mins one mouse was injected in order to further collect enough blood sample, and tail blood samples were collected at −60 or −45 min, just before glucose (0 min), and 15 and 30 min after glucose injection, in EDTA-coated tubes, centrifuged at 4°C, 2000 ***g*** for 10 min and the obtained plasma stored at −20°C until analysis. Plasma insulin was quantified by ELISA kit (Mercodia) following the manufacturer's instructions. Glucose area under the curve (AUC) was calculated using the open software ImageJ.

### Statistical analyses

Data are expressed as mean±s.e.m. Statistical analyses were performed using GraphPad Prism Software (San Diego, CA, USA). Comparisons were made using one-way ANOVA analysis for *in vitro* experiments and repeated measures ANOVA for GTTs. Bonferroni's post-hoc test was used. A probability level <0.05 was considered statistically significant.
